# Terror and bliss? Commonalities and distinctions between sleep paralysis, lucid dreaming, and their associations with waking life experiences

**DOI:** 10.1111/jsr.12441

**Published:** 2016-07-27

**Authors:** Dan Denis, Giulia L. Poerio

**Affiliations:** ^1^ Department of PsychologyUniversity of SheffieldSheffieldUK; ^2^ Department of PsychologyUniversity of YorkYorkUK

**Keywords:** anomalous sleep experiences, parasomnia, REM dissociation, wake–sleep continuum

## Abstract

Sleep paralysis and lucid dreaming are both dissociated experiences related to rapid eye movement (REM) sleep. Anecdotal evidence suggests that episodes of sleep paralysis and lucid dreaming are related but different experiences. In this study we test this claim systematically for the first time in an online survey with 1928 participants (age range: 18–82 years; 53% female). Confirming anecdotal evidence, sleep paralysis and lucid dreaming frequency were related positively and this association was most apparent between lucid dreaming and sleep paralysis episodes featuring vestibular‐motor hallucinations. Dissociative experiences were the only common (positive) predictor of both sleep paralysis and lucid dreaming. Both experiences showed different associations with other key variables of interest: sleep paralysis was predicted by sleep quality, anxiety and life stress, whereas lucid dreaming was predicted by a positive constructive daydreaming style and vividness of sensory imagery. Overall, results suggest that dissociative experiences during wakefulness are reflected in dissociative experiences during REM sleep; while sleep paralysis is related primarily to issues of sleep quality and wellbeing, lucid dreaming may reflect a continuation of greater imaginative capacity and positive imagery in waking states.

## Introduction

This paper examines the unexplored similarities and differences between sleep paralysis and lucid dreaming and their associations with waking states of consciousness (e.g. daydreaming, dissociative experiences), wellbeing and beliefs. Sleep paralysis is a period of inability to perform voluntary movements at either sleep onset or upon awakening (American Academy of Sleep Medicine, 2014). Episodes are often accompanied by a wide range of bizarre hallucinations comprising three categories (Cheyne, 2003; Cheyne *et al*., 1999): intruder hallucinations, which involve a sense of an evil presence and multi‐sensory hallucinations of an intruder; incubus hallucinations, characterized by the feeling of pressure on the chest, suffocation and physical pain; and vestibular‐motor (V‐M) hallucinations, which feature illusory‐movement and out‐of‐body experiences. Intruder and incubus hallucinations typically co‐occur and are accompanied by fear, whereas V‐M hallucinations are more positive, involving feelings of bliss (Cheyne, 2003).

Lucid dreaming is a dream involving awareness of dreaming (Schredl and Erlacher, 2004) and is characterized by increased insight, control, access to waking memories, dissociation from one's own body, logical thought, and more positive emotion (compared to non‐lucid dreaming) (Voss *et al*., 2013). Anecdotally, sleep paralysis and lucid dreaming are thought to be related, with accounts of people entering sleep paralysis directly from a lucid dream and vice versa (Emslie, 2014). It is also likely that these sleep experiences are underlined by similar neurophysiology (Dresler *et al*., 2012; Terzaghi *et al*., 2012; Voss *et al*., 2009), and both can be conceptualized as dissociated rapid eye movement (REM) states (i.e. that aspects of waking consciousness are present during REM) (Mahowald and Schenck, 2005).

Despite suggestions for an overlap, research has not yet explored their co‐occurrence and similarities. Although there is reason to suspect their close association (e.g. in terms of frequency), sleep paralysis and lucid dreaming show differences depending on their specific characteristics, which leads to predictions about how they might be different as well as similar. For example, because lucid dreaming is typically positively valenced while sleep paralysis is overwhelmingly negative, we might expect stronger positive associations between lucid dreams and sleep paralysis characterized by V‐M hallucinations (compared to intruder/incubus hallucinations). Another distinction is that sleep paralysis involves full return to wakefulness during REM‐induced muscle atonia, whereas lucid dreaming involves the recovery of aspects of consciousness experienced during waking while the person remains asleep (in REM).

Sleep paralysis and lucid dreaming appear to be similar but different experiences, and we sought to characterize their commonalities and distinctions by examining their patterns of associations with variables known, or suggested, to be related to either or both experience. Specifically, we assessed the unique predictors of sleep paralysis and lucid dreaming to pinpoint variables that are associated with both experiences, and those which relate to only one or the other. We chose a number of predictors in four categories (sleep; waking experiences; wellbeing; beliefs) based on previous research and theory linking variables with either or both sleep paralysis and lucid dreaming. Crucially, however, this is the first study, to our knowledge, to examine these variables in relation to both experiences.

Sleep quality was assessed due to its known relationship with sleep paralysis (Denis *et al*., 2015) and because sleep disruption can induce sleep paralysis episodes (Takeuchi *et al*., 1992, 2002). We examined waking state experiences relevant to both experiences. Daydreaming frequency and style were assessed due to suggestions that dreaming and daydreaming share similarities, such as their association with the same neural networks (Domhoff and Fox, 2015; Fox *et al*., 2013). Dissociative experiences were examined because previous research has associated uncontrollable and negative sleep experiences (e.g. sleep paralysis) with more severe daytime dissociative experiences but not between dissociative experiences and lucid dreaming (Giesbrecht and Merckelbach, 2006; van der Kloet *et al*., 2012; Watson, 2001). Trait mindfulness was measured to serve as a parallel for lucidity in waking life experiences and was expected to play an opposite role to dissociative experiences. We also examined individual differences in waking sensory imagery to explore whether this translated to greater vividness of the hallucinatory content of sleep paralysis and lucid dreams.

Wellbeing measures were examined (depression, anxiety and life stress) due to their known associations with sleep paralysis (Denis *et al*., 2015; Ramsawh *et al*., 2008; Szklo‐Coxe *et al*., 2007) but unexplored associations with lucid dreaming. Finally, we examined paranormal beliefs and conspiratorial thinking, because some paranormal experiences are believed to be due to sleep paralysis (e.g. space alien abduction) (French *et al*., 2008) and because lucid dreaming has often been linked with the belief in astral projection (Irwin, 1988).

## Method

### Participants

A total of 1928 participants took part [mean_age_ = 34.17, standard deviation (SD): 13.62, range: 18–82 years, 53% female]. Participants were invited to take part in an online survey through advertisements on a university mailing list (i.e. students and staff at the University of Sheffield interested in taking part in research), and on lucid dreaming and sleep paralysis websites and forums (these are listed in the Acknowledgements section). The study was described as an investigation into the links between people's experiences of wakefulness and sleep. Participants were asked to indicate whether they had been diagnosed with any of the following: narcolepsy, epilepsy, post‐traumatic stress disorder, panic disorder, anxiety disorder or depression. They were also asked to indicate if they had experienced sexual and/or physical abuse. Thirty‐four participants (1.5%) had experienced at least one of the above, and were excluded from further analyses.

### Measures

#### Sleep

##### Sleep paralysis

This was measured using the 42‐item Waterloo Unusual Sleep Experiences Questionnaire–VIIa (WQ; Cheyne, 2002). Participants indicated the frequency of sleep paralysis on a seven‐point scale (0: never; 1: once; 2: several times in life; 3: several times a year; 4: monthly; 5: weekly; 6: several times a week) and the intensity/vividness of this experience from 1 (vague and suggestive, a hint of something) to 7 (a very clear and distinct impression, as clear as any everyday experience), with the exception that if sleep paralysis was never experienced, then intensity was scored automatically as 0. Scores for sleep paralysis frequency and intensity were averaged to form separate scores (possible ranges were from 0 to 6 and 0 to 7, respectively). Participants who indicated experiencing sleep paralysis then indicated the frequency (0: never; 1: occasionally; 2: frequently; 3: always) and intensity (0–7, as above) of three types of hallucinations during sleep paralysis. Three subscales indexed intruder (five items, e.g. ‘During the experience I imagined that I saw a something: a shape, person or being of some kind’: *α*_frequency_ = 0.78; *α*_intensity_ = 0.78), incubus (four items, e.g. ‘During the experience I felt pressure on my chest or other part(s) of my body’: *α*_frequency_ = 0.75; *α*_intensity_ = 0.78) and V‐M (eight items, e.g. ‘During the experience I had a sensation of floating’: *α*_frequency_ = 0.81; *α*_intensity_ = 0.85) hallucinations. Items for each subscale were averaged to provide a separate score for each of the three hallucination types for both frequency and intensity; possible scores for frequency and intensity for each subscale ranged from 0 to 3 and 0 to 7, respectively.

##### Lucid dreaming

Participants indicated their frequency of lucid dreaming (‘During lucid dreaming, one is—while dreaming—aware of the fact that one is dreaming. It is possible to deliberately wake up or control the dream action or to observe passively the course of the dream with this awareness. How frequently do you experience lucid dreams?’) on a scale from 0 (never) to 7 (several times a week) (Schredl and Erlacher, 2004). Participants also indicated their dream recall frequency (‘Please rate how frequently you can recall dreams’) on a scale from 0 (never) to 7 (almost every morning). Possible scores for each of these items ranged from 0 to 7; items were kept separate for analyses.

##### Sleep quality

This was measured with the eight‐item Sleep Condition Indicator (SCI; Espie *et al*., 2014). Participants considered a typical night in the past month and rated various aspects of their sleep, including sleep onset (‘How long does it take you to fall asleep?’); waking during sleep (‘If you wake up during the night… how long are you awake for in total?’); perceived sleep quality (‘How would you rate your sleep quality?’); and the effect of poor sleep on various aspects of life (e.g. ‘To what extent has poor sleep affected your mood, energy, or relationships?’). Response scales ranged from 0 to 4, but differed depending on the question. Items were summed to create an overall sleep condition score with possible values ranging from 0 to 32 (*α* = 0.86); lower scores indicate poorer sleep quality.

#### Waking state experiences

##### Daydreaming frequency

This was measured using the 12‐item Daydreaming Frequency Scale (DDFS; Singer and Antrobus, 1972). Participants rated their daydreaming frequency in general and during a variety of situations (Giambra, 1993). Response options differ among items, but each item is rated on a five‐point scale with greater values indicating greater daydreaming frequency. Items were summed to provide a score for daydreaming frequency with possible values from 12 to 60; higher scores indicate a greater level of daydreaming activity in daily life (*α* = 0.94).

##### Positive constructive daydreaming

This was measured using the 15‐item Positive Constructive Daydreaming scale (PCDD; Singer and Antrobus, 1972). Participants rated the extent to which 15 statements about daydreaming applied to them (e.g. ‘A really original idea can sometimes develop from a really fantastic daydream’) from 1 (strongly uncharacteristic) to 5 (strongly characteristic). Negatively worded items were reverse‐scored and items were averaged to provide a score for positive constructive daydreaming with possible values from 1 to 5; higher scores indicate more positive attitudes towards, and outcomes of, daydreaming (*α* = 0.85).

#### Dissociative experiences

This was measured using the 28‐item Dissociative Experiences Scale‐II (DES‐II; Carlson and Putnam, 1993). Participants rated the percentage of time occupied by dissociative experiences over the past month (e.g. ‘Finding yourself in a place and having no idea how you got there’) using 100‐point sliding scales. Scores for each item were summed to create and overall score with possible values from 0 to 2800; higher scores indicate greater dissociative experiences (*α* = 0.93).

#### Mindfulness

Trait mindful attention was measured with the 15‐item Mindful Attention Awareness Scale (MAAS; Brown and Ryan, 2003). Participants rated the extent to which they experience paying attention to their present environment (e.g. ‘I find myself doing things without paying attention’) from 1 (almost always) to 6 (almost never). Items were averaged to create an overall score with possible values from 1 to 6; higher values indicate less dispositional mindful attention (*α* = 0.88).

#### Imagery

This was measured using the 35‐item Plymouth Sensory Imagery Questionnaire (Psi‐Q; Andrade *et al*., 2013) Participants were given five cues to generate sensory imagery (e.g. ‘Imaging the appearance of… a sunset’) in seven modalities: visual, auditory, smell, taste, touch, bodily sensations and emotions. They then rated the vividness of their mental imagery from 0 (no image at all) to 10 (as vivid as real life). Scores were averaged with higher scores (possible values ranging from 0 to 10) indicating greater self‐reported imagery across sensory modalities (*α* = 0.98).

#### Wellbeing

##### Depression

Depressed mood was measured with the 13‐item Moods and Feelings Questionnaire (MFQ; Angold *et al*., 1995). Participants rated the extent to which they had felt or acted during the past 2 weeks (e.g. ‘I felt miserable and unhappy’) on three‐point scales (not true, sometimes, true). Items were summed to create an overall score for depression with possible values from 13 to 39; higher scores indicate greater depressive symptoms (*α* = 0.90).

##### Anxiety

Trait anxiety was measured using the 20‐item trait version of the State‐Trait Anxiety Inventory (STAI; Spielberger *et al*., 1983). Participants rated the extent to which they generally feel (e.g. ‘I make decisions easily’) from 1 (almost never) to 4 (almost always). Positive items were reverse‐scored and items were summed with possible values from 20 to 80; higher scores indicate greater trait anxiety (*α* = 0.94).

##### Life stress

This was measured using the 10‐item Perceived Stress Scale (PSS; Cohen *et al*., 1983). Participants rated the extent to which they had felt and thought in a certain way during the past month (e.g. ‘How often have you felt confident about your ability to handle your personal problems?’) from 1 (never) to 5 (very often). Positively worded items were reverse‐scored and items were summed with possible values from 5 to 50; higher scores indicate greater perceived stress during the past month (*α* = 0.90).

#### Beliefs

##### Conspiracy beliefs

This was measured using the 15‐item Generic Conspiracist Beliefs Scale (GCBS; Brotherton *et al*., 2013). Participants rated their endorsement of typical conspiracy beliefs (e.g. ‘The government is involved in the murder of innocent citizens and/or well‐known public figures, and keeps this a secret’) from 1 (definitely not true) to 5 (definitively true). Items were averaged with possible values from 1 to 5; higher scores indicate greater belief in conspiracy theories (*α* = 0.95).

##### Paranormal beliefs

This was measured using the 26‐item revised Paranormal Belief Scale (PBS; Tobacyk, 2004). Participants rated their agreement with paranormal beliefs [e.g. ‘Some individuals are able to levitate (lift) objects through mental forces’] from 1 (strongly disagree) to 7 (strongly agree). The items ‘Mindreading is not possible’ and ‘There is life on other planets’ were removed, because they compromised internal scale reliability. Scores were summed with possible values from 26 to 182; higher scores indicate greater paranormal beliefs (*α* = 0.95).

### Procedure

The survey was administered using the survey platform Qualtrics. Participants read an information sheet, provided informed consent and entered their demographics. Given our focus on sleep experiences, participants first completed sleep measures (sleep paralysis, lucid dreaming and sleep quality) in random order followed by waking state, wellbeing and belief measures (also presented randomly). The study received ethical approval from the University of Sheffield Department of Psychology Ethics Committee.

### Statistical analyses

Predictors of sleep paralysis and lucid dreaming were analysed using multiple linear regression. Some dependent variables were distributed non‐normally, so analyses were run on both the original and transformed data. Results with non‐transformed scores are reported, as few differences were observed. Only variables showing significant correlations with the dependent variables were entered into the regressions. As dream recall frequency is known to be associated highly with lucid dreaming frequency (Watson, 2001), dream recall was controlled for in all analyses of lucid dreaming.

Additionally, measures of sleep paralysis and lucid dreaming frequency were dichotomized into low and high sleep paralysis/lucid dreaming using a median split procedure. We then ran a series of independent *t*‐tests to examine differences between low and high frequency experiencers of sleep paralysis and lucid dreaming in average levels of our variables of interest. This complementary approach was taken to also allay concerns regarding non‐normality in the regression analyses; results are provided in the Supporting information.

## Results

### Descriptive statistics

**Figure 1:**
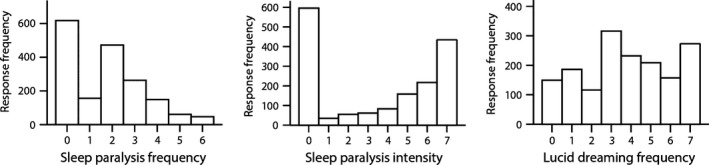
Histograms showing the distribution of sleep paralysis frequency, sleep paralysis intensity, and lucid dreaming frequency in the current sample. For sleep paralysis frequency, 0: never; 1: once; 2: several times in life; 3: several times a year; 4: monthly; 5: weekly; 6: several times a week. For sleep paralysis intensity, 0: not applicable, 1 (vague and suggestive, a hint of something) to 7 (a very clear and distinct impression, as clear as everyday experience). For lucid dreaming frequency, 0: never, 1: less than once a month, 2: about once a month, 3: twice or three times a month, 4: about once a week, 5: several times a week, 6: almost every morning.

Of the sample, 64 and 91% had experienced sleep paralysis and lucid dreaming at least once in their lives. The distribution of frequency and intensity of episodes are displayed in Fig. 1. This proportion is probably biased due to the recruiting strategy. The means, standard deviations and intercorrelations of study variables are presented in Table 1. Means and standard deviations of sleep paralysis hallucinations and their correlations with predictor variables are displayed in Table 2.

**Table 1:** Correlations between sleep paralysis, lucid dreams and predictor variables

		M	SD	1	2	3	4	5	6	7	8	9	10	11	12
1	SP frequency	1.70	1.61												
2	LD frequency	3.74	2.21	0.15***											
3	Sleep quality	19.77	7.87	−0.18***	−0.02										
4	Daydreaming frequency	37.08	11.23	0.08**	0.09***	−0.15***									
5	Positive constructive daydreaming	3.40	0.67	0.09**	0.25***	0.03	0.49***								
6	Dissociative experiences	458.43	416.91	0.16***	0.18***	−0.25***	0.37***	0.20***							
7	Mindfulness	3.89	0.85	−0.14***	0.04	0.33***	−0.39***	−0.09**	−0.45**						
8	Imagery	7.47	1.98	0.05	0.19***	0.05	0.05	0.27***	0.09**	0.09**					
9	Depression	19.84	5.90	0.15***	−0.06*	−0.45***	0.29***	−0.06*	0.38***	−0.44***	−0.09**				
10	Anxiety	43.70	12.28	0.11***	−0.09**	−0.47***	0.29***	−0.11***	0.31***	−0.49***	−0.09**	0.76***			
11	Stress	17.21	7.60	0.15***	−0.09**	−0.42***	0.26***	−0.08**	0.28***	−0.46***	−0.12***	0.74***	0.81***		
12	Conspiracy beliefs	2.61	0.93	0.04	0.12***	−0.05	0.13***	0.10***	0.28***	−0.06*	0.13***	0.15***	0.10***	0.10***	
13	Paranormal beliefs	63.15	31.02	0.06*	0.13***	−0.01	0.10***	0.16***	0.22***	0.00	0.18***	0.03	−0.01	0.00	0.60***

M, mean; SD, standard deviation; SP, sleep paralysis; LD, lucid dreaming.

***P < 0.001; **P < 0.01; *P < 0.05.

**Table 2:** Sleep paralysis hallucinations correlated with predictor variables

	M	SD	1	2	3	4	5	6	7	8	9	10	11
Intruder frequency	0.88	0.72	−0.16***	0.11**	0.09*	0.20***	−0.10**	0.10**	0.15***	0.13***	0.15***	0.14***	0.20***
Intruder intensity	3.02	2.12	−0.10**	0.08*	0.10**	0.09*	−0.04	0.13**	0.08*	0.05	0.05	0.13**	0.17***
Incubus frequency	0.74	0.72	−0.19***	0.08*	0.02	0.19***	−0.13***	0.05	0.21***	0.19***	0.20***	0.04	0.01
Incubus intensity	2.66	2.22	−0.16***	0.04	0.03	0.13**	−0.10**	0.09*	0.15***	0.14***	0.13**	0.09*	0.11**
V‐M frequency	0.64	0.56	−0.06	0.10**	0.16***	0.28***	−0.09*	0.12**	0.13***	0.04	0.04	0.18***	0.23***
V‐M intensity	2.41	1.92	−0.04	0.03	0.17***	0.17***	−0.01	0.15***	0.04	−0.05	−0.07	0.17***	0.20***

M, mean; SD, standard deviation; V‐M, vestibular‐motor; 1, sleep quality; 2, daydreaming frequency; 3, positive constructive daydreaming; 4, dissociative experience; 5, mindfulness; 6, imagery; 7, depression, 8, anxiety, 9, life stress; 10, conspiracy beliefs; 11: paranormal beliefs.

***P < 0.001; **P < 0.01; *P < 0.05.

### Are sleep paralysis and lucid dreaming frequency associated?

Sleep paralysis and lucid dreaming frequency were correlated significantly positively, *r = *0.15, *P < *0.001. Lucid dreaming frequency was also correlated significantly positively with intruder and V‐M sleep paralysis hallucinations (intruder: frequency, *r = *0.08, *P = *0.01; intensity, *r = *0.10, *P = *0.01, V‐M: frequency, *r* = 0.25, *P < *0.001, intensity, *r = *0.28, *P < *0.001) but not incubus hallucinations (frequency, *r = *−0.004, *P = *0.89, intensity, *r = *0.03, *P = *0.41). Considering both intruder and V‐M hallucinations frequency and intensity as predictors of lucid dreaming in a multiple regression, only V‐M hallucination intensity predicted lucid dreaming frequency significantly; *β* = 0.36, 95% confidence interval (CI): 0.03–0.68. The overall regression model was significant; *F*_(4, 747)_ = 48.97, *P < *0.001, *R* ^2^ = 0.24.

### Common and distinct predictors of sleep paralysis and lucid dreaming

**Figure 2:**
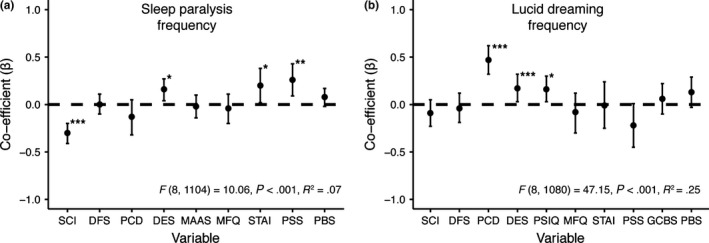
Predictors of sleep paralysis and lucid dreaming frequency. SCI, sleep quality; DFS, daydreaming frequency; PCD, positive constructive daydreaming; DES, dissociative experiences; MAAS, mindfulness; PSIQ, imagery; MFQ, depression; STAI, anxiety; PSS, life stress; GCBS, conspiracy beliefs; PBS, paranormal beliefs. (a) Predictors of sleep paralysis frequency; (b) predictors of lucid dreaming frequency, after dream recall frequency had been controlled for. Error bars indicate 95% confidence intervals. ***P < 0.001, **P < 0.01; *P < 0.05.

Predictors of sleep paralysis frequency are displayed in Fig. 2a. Sleep quality, dissociative experiences, anxiety and stress were all independent predictors of sleep paralysis. Predictors of lucid dreaming frequency are displayed in Fig. 2b. Positive constructive daydreaming, dissociative experiences and imagery were all independent predictors of lucid dreaming.

**Figure 3:**
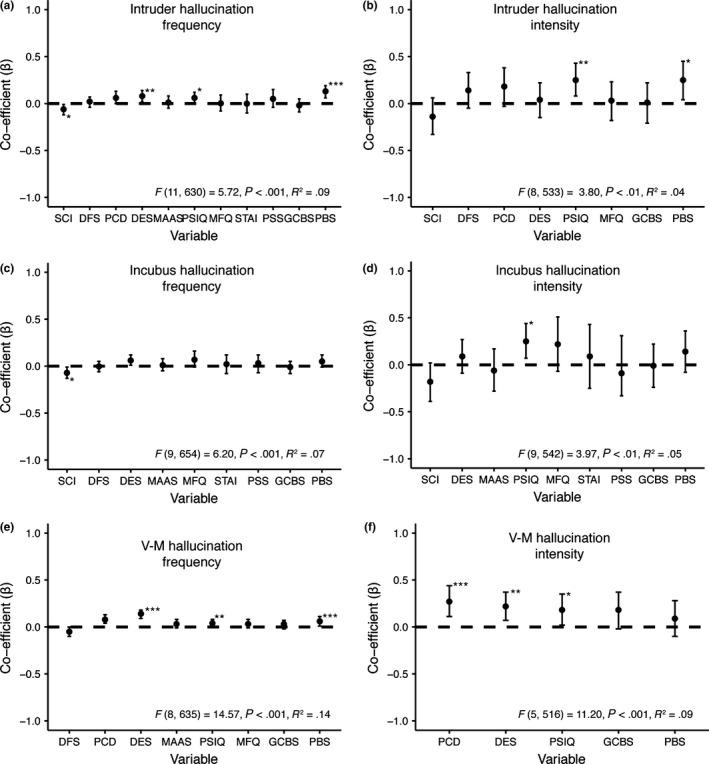
Predictors of sleep paralysis hallucination frequency/intensity. SCI, sleep quality; DFS, daydreaming frequency; PCD, positive constructive daydreaming; DES, dissociative experiences; MAAS, mindfulness; PSIQ, imagery; MFQ, depression; STAI, anxiety; PSS, life stress; GCBS, conspiracy beliefs; PBS, paranormal beliefs. (a) Predictors of intruder hallucination frequency; (b) predictors of intruder hallucination intensity; (c) predictors of incubus hallucination frequency; (d) predictors of incubus hallucination intensity; (e) predictors of V‐M hallucination frequency; (f) predictors of V‐M hallucination intensity. Error bars indicate 95% confidence intervals. ***P < 0.001; **P < 0.01; *P < 0.05.

Independent multiple regressions predicting each sleep paralysis hallucination type are displayed in Fig. 3a–f. For intruder hallucination frequency; sleep quality, dissociative experiences and paranormal belief were all independent predictors (Fig. 3a). Imagery and paranormal beliefs predicted intruder hallucination intensity (Fig. 3b). Sleep quality was the only significant predictor of incubus hallucination frequency, although dissociative experiences was a marginally significant predictor; *P = *0.052 (Fig. 3c). For incubus hallucination intensity; imagery was the only significant predictor (Fig. 3d). V‐M hallucination frequency was predicted by daydreaming frequency, positive constructive daydreaming, dissociative experiences, imagery and paranormal beliefs (Fig. 3e). Finally; V‐M hallucination intensity was predicted significantly by positive constructive daydreaming, dissociative experiences and imagery (Fig. 3f).

## Discussion

This large‐scale online survey examined the relationship between sleep paralysis and lucid dreaming, and associations with other waking state variables, including measures of daydreaming, imagery, dissociative experiences, wellbeing and unusual beliefs. For the first time we sought to characterize commonalities and distinctions between these two experiences by examining their unique and shared predictors.

Consistent with anecdotal reports (Emslie, 2014) and theoretical accounts of their neurophysiology (Terzaghi *et al*., 2012; Voss *et al*., 2009), the frequency of sleep paralysis and lucid dreaming were correlated positively, indicating the common co‐occurrence of these sleep experiences. In particular, lucid dreaming was associated positively with sleep paralysis featuring intense V‐M hallucinations (as opposed to intruder and incubus hallucinations). This suggests that both experiences are REM dissociated states characterized by positive emotion (Cheyne, 2003; Voss *et al*., 2013). Additionally, both V‐M hallucination intensity and frequency, and lucid dreaming frequency were predicted positively by a positive constructive daydreaming style, which reflects a positive and playful attitude towards waking imagery (Singer and Antrobus, 1972). One possibility is that the relationship between V‐M experiences in sleep paralysis and lucid dreaming, and their unique but common connection with positive constructive daydreaming, may be underlined by the more general personality trait of openness to experience. This is a personality trait characterized by curiosity, sensitivity and an exploration of ideas, feelings and sensations that has been associated reliably with positive constructive daydreaming (Zhiyan and Singer, 1997). The idea that night‐ and daytime experiences are affected by personality traits (especially openness to experience) fits well with research linking ‘thin boundaries’ with unusual sleep experiences (Hartmann, 1992).

Dissociative experience was the only common predictor of both sleep paralysis and lucid dreaming frequency, indicating that individuals who experience both unusual sleep experiences also experience greater dissociative experiences in daily life. This fits well with research and theory suggesting that dissociative experiences are fuelled by sleep disturbances (van der Kloet *et al*., 2012), but this study is the first, to our knowledge, to link lucid dreaming to dissociative experiences (Koffel and Watson, 2009). Given the associations between lucid dreaming frequency, V‐M hallucinations, daydreaming variables and dissociative experiences, future research could examine the dynamic interactions between unusual sleep experiences, daydreaming and dissociative experiences, particularly within clinical populations (e.g. in the dissociative disorders). Experience‐sampling methodology (involving sampling sleep and waking experiences repeatedly as they unfold in ecologically valid settings) would be ideally suited to examining temporal associations between variables of interest and will help to untangle cause from consequence. The overlap between waking and sleeping states of dissociation (daydreaming and dissociated REM experiences, respectively) and their mutual influence may shed light on the development and maintenance of clinical disorder, in particular dissociation (for a recent example of this approach in a clinical case study of depersonalization/derealization, see Poerio *et al*., 2016).

Interesting distinctions between sleep paralysis and lucid dreaming also emerged, indicating potentially different causes, consequences or concomitants. Sleep paralysis frequency (but not lucid dreaming) was associated with poorer sleep quality and greater stress and anxiety, whereas lucid dreaming frequency (but not sleep paralysis) was associated with positive constructive daydreaming and more vivid imagination. One possibility is that stress and anxiety may fuel and exacerbate episodes of sleep paralysis, possibily by causing sleep disruption. Alternatively, sleep paralysis may have a detrimental effect on wellbeing and sleep quality, because it can be a terrifying and unwanted experience that has negative downstream consequences on psychosocial functioning (Sharpless *et al*., 2010). In contrast, lucid dreaming was not associated with negative affective states or poor sleep quality, and may be reflective of a continuation of greater imaginative capacity and positive relationship with imagery in waking states. Future research is required to characterize temporal associations between wellbeing and sleep and the relative strength of bi‐directional effects (e.g. is previous stress a stronger predictor of sleep paralysis occurrence than sleep paralysis is of later stress?).

Mirroring the association between lucid dreaming and imaginative capacity, the intensity (but not frequency) of all types of sleep paralysis hallucinations were associated positively with imaginary capacity. One implication of this is that individual differences in imagination are a double‐edged sword, having both a positive and negative impact on sleep experiences. Whereas a greater imaginative capacity may be associated with more frequent lucid dreaming (a typically positive experience associated with bliss), it may also be associated with more vivid experiences of terror in sleep paralysis intruder/incubus hallucinations.

These results should be considered alongside a number of limitations. First, as noted previously, the cross‐sectional nature of this study prevents any definitive conclusions about the causal nature of the observed associations. It also does not rule out any third variable explanations for the findings. For example, although we found that dissociative experiences predicted both sleep paralysis and lucid dreaming frequency, it is also possible that sleep paralysis and lucid dreaming are both simply manifestations of dissociative experiences, or reflect predispositions to experience dissociation at the trait level. Secondly, we should note that the *R* ^2^ values for some of our regression models explained a relatively small (but statistically significant) proportion of the variance (0.04–0.25). Therefore, the impact of some of our findings (particularly for models predicting sleep paralysis hallucinations) should be considered tentatively. Thirdly, although our online survey was well suited for recruiting a large and diverse sample with unique sleep experiences, it did not allow us to ensure that participants were optimally responding (e.g. honestly and conscientiously), as might occur in laboratory settings.

Notwithstanding these limitations, this study has provided the first evidence linking sleep paralysis and lucid dreaming, not only to each other but also to important and relevant waking states such as daydreaming, dissociative experiences and affect. Although the correlational and retrospective nature of this investigation prevents causal interpretation, our findings provide an exciting starting point for future research in this area. Moving forward, we recommend building on the observed associations between dissociations in waking and sleeping states using intensive longitudinal methods. Such nomothetic studies could examine dynamic relationships and interactions between the variables measured here to characterize accurately how these experiences unfold in daily life both within and between individuals. Ultimately, this future work may provide a useful and nuanced evidence base to inform interventions aimed at dampening the terror and enhancing the bliss associated with sleep paralysis and lucid dreaming, respectively.

## Conflict of Interest

Both authors declare no conflicts of interest.

## Author Contributions

Both authors designed the study, collected and analysed the data and wrote the manuscript.

## Supporting information


**Data S1.** Median split analyses.
**Table S1.** Means and standard deviations of predictor variables in low and high sleep paralysis/lucid dreaming categories for median split analysis
