# Supplementary material for: Terror and bliss? Commonalities and distinctions between sleep paralysis, lucid dreaming, and their associations with waking life experiences
Source: J Sleep Res. 2016 Jul 27;26(1):38–47. doi: 10.1111/jsr.12441 (PMC5245115; doi:10.1111/jsr.12441)
Supplement: Supplementary file 1 — Data S1. Median split analyses. Table S1. Means and standard deviations of predictor variables in low and high sleep paralysis/lucid dreaming categories for median split analysis [file JSR-26-38-s001.docx]

Supporting information

Median split analyses

Median split categorised participants as low or high frequency experiencers of sleep paralysis (low (n = 772): *M* = .20, *SD* = .40; high (n = 960): *M* = 2.92, *SD* = 1.12) and lucid dreaming (low (n = 831): *M* = 1.79, *SD* = 1.18; high (n = 901): *M* = 5.53, *SD* = 1.18). See Table S1 for more detail.

High (compared to low) frequency sleep paralysis experiencers had significantly poorer sleep quality (*t*(1591) = 5.87, *p* < .001), reported daydreaming more frequently (*t*(1374) = -2.15, *p* = .032), had a more positive-constructive daydreaming style (*t*(1318) = -3.08, *p* = .002), experienced greater dissociation (*t*(1163) = -3.02, *p* = .003), greater mindfulness (*t*(1336) = 4.05, *p* < .001), higher levels of depression (*t*(1357) = -3.72, *p* < .001), anxiety (*t*(1361) = -2.58, *p* = .010), and stress (*t*(1371) = -4.39, *p* < .001), and stronger paranormal beliefs (*t*(1349) = -2.13, *p* = .033). High and low frequency experiencers of sleep paralysis did not show significant differences in their levels of imagery (*t*(1340) = -1.02, *p* = .308) or belief in conspiracy theories (*t*(1353) = -.95, *p* = .342).

Mirroring the results for sleep paralysis, high (compared to low) frequency lucid dreamers reported daydreaming more frequently (*t*(1375) = -3.56, *p* < .001), had a more positive-constructive daydreaming style (*t*(1319) = -8.29, *p* < .001), experienced greater dissociation (*t*(1168) = -4.17, *p* < .001), and had stronger paranormal beliefs (*t*(1350) = -3.12, *p* = .002). However, unlike sleep paralysis experiencers, high (compared to low) frequency lucid dreamers also had significantly more vivid imagery (*t*(1254) = -6.07, *p* < .001), were *less* stressed *t*(1372) = 2.37, *p* = .018), and marginally *less* anxious (*t*(1362) = 1.80, *p* = .071), and reported greater belief in conspiracy theories *t*(1354) = -3.26, *p* = .001). High and low frequency lucid dreamers did not show significant differences in their sleep quality (*t*(1601) = 1.21, *p* = .226), levels of mindfulness (*t*(1359) = -1.12, *p* = .263). or depression (*t*(1367) = 1.28, *p* = .203).

Table S1. Means and standard deviations of predictor variables in low and high sleep paralysis/lucid dreaming categories for median split analysis

|  | Sleep Paralysis | *M* | *SD* | Lucid Dreaming | *M* | *SD* |
| --- | --- | --- | --- | --- | --- | --- |
| Sleep Quality | Low | 21.05 | 7.64 | Low | 20.02 | 7.86 |
|  | High | 18.74 | 7.96 | High | 19.54 | 7.96 |
| Daydreaming Frequency | Low | 36.36 | 11.26 | Low | 35.93 | 11.08 |
|  | High | 37.67 | 11.17 | High | 38.09 | 11.27 |
| Positive Constructive Daydreaming | Low | 3.34 | 0.67 | Low | 3.24 | 0.65 |
|  | High | 3.45 | 0.67 | High | 3.54 | 0.66 |
| Dissociation | Low | 419.13 | 389.64 | Low | 404.42 | 380.03 |
|  | High | 492.20 | 436.17 | High | 504.58 | 441.09 |
| Mindfulness | Low | 3.99 | 0.82 | Low | 3.86 | 0.82 |
|  | High | 3.81 | 0.86 | High | 3.92 | 0.87 |
| Imagery | Low | 7.41 | 1.96 | Low | 7.12 | 2.07 |
|  | High | 7.52 | 1.99 | High | 7.77 | 1.84 |
| Depression | Low | 19.20 | 5.60 | Low | 20.05 | 6.00 |
|  | High | 20.37 | 6.09 | High | 19.65 | 5.80 |
| Anxiety | Low | 42.78 | 11.90 | Low | 44.35 | 12.10 |
|  | High | 44.50 | 12.55 | High | 43.15 | 12.41 |
| Stress | Low | 16.24 | 7.37 | Low | 17.73 | 7.60 |
|  | High | 18.03 | 7.71 | High | 16.76 | 7.59 |
| Conspiracy Beliefs | Low | 2.58 | 0.93 | Low | 2.52 | 0.90 |
|  | High | 2.63 | 0.92 | High | 2.68 | 0.95 |
| Paranormal Beliefs | Low | 61.17 | 29.95 | Low | 60.32 | 30.56 |
|  | High | 64.78 | 31.80 | High | 65.58 | 31.22 |
